# The Surgical Treatment Required to Prepare the Alveolar Process and Gum Tissues for Prosthetic Restorations*Extract from a paper read before the National Society of Denture Prosthetists at Milwaukee, and printed in the February, 1922, *Journal N. D. A*.

**Published:** 1922-03

**Authors:** W. A. Giffen, Lloyd Rogers, J. W. Kemper


### THE SURGICAL TREATMENT REQUIRED TO PREPARE THE ALVEOLAR PROCESS AND GUM TISSUES FOR PROSTHETIC RESTORATIONS *

BY W. A. GIFFEN, LLOYD ROGERS AND J. W. KEMPER

When we consider how small the degree of efficiency of artificial dentures is, compared with the natural masticatory apparatus; and when we think of the difficulties encountered to obtain this small result, even under normal circumstances, we realize that the importance of surgical interference to correct any abnormality of the maxillae can not be too greatly emphasized. The dentist no longer finds it necessary to consider these irregularities of the alveolar ridge, permanent or irremovable barriers around which he must construct his dentures, but much to the contrary—he plans the surgical shaping of the arches as the builder clears ground for the construction of his foundation.

Just in recent years has this branch of surgery gained prominence. A simple technic has been worked out with such success from both aesthetic and efficiency standpoints

---

* Extract from a paper read before the National Society of Denture Prosthetists at Milwaukee, and printed in the February, 1922, *Journal N. D. A.*

that it is fast becoming the routine practice of the average prosthodontist.

It is the purpose of this paper to give the various steps in the operation, paying special attention to the curettage of septic sockets and enumeration of some of the dangers encountered in this work.

The patient is prepared as in the case of any major operation and all rules of asepsis strictly adhered to during the operation. Conductive or general anesthesia under nitrous-oxide-oxygen is used. We prefer general anesthesia.

Before the extraction of any of the teeth, the buccal and labial flaps of soft tissue are prepared. A very even flap is had by making an incision starting at the posterior tooth and continuing across the ridge in as nearly a straight line as possible, meeting the highest point on the face of the crown of each tooth, excluding the gum festoons from the flap. In spaces where teeth have been previously extracted, the incision is carried across the ridge to the next tooth. This affords the longest possible flap (which is usually needed) and if it comes at all close to the lingual ridge of soft tissue, a very neat result is realized, with quick healing and very little discomfort to the patient. By means of the periosteal elevators, the muco-periosteum is retracted, exposing the whole of the alveolar ridge. The teeth are then extracted with the least destruction of the buccal and labial plate of the alveolar ridge. Without mutilation of the ridge there is less shock to the patient, the arch is easier to shape, whereby the possibilities of ideal results and more rapid healing are increased. The method of procedure for the removal of the teeth will of course depend upon the case.

The mouth now presents a mutilated and ugly appearance with the gum festoons still attached to the interproximal osseous septae. These festoons are dissected loose from those bony septae back to the lingual ridge and by means of surgical shears, following close to the lingual ridge of the arch, are excised as closely as possible, allowing no lingual ridge of soft tissue to remain. The pointed spiculae of bone between the root sockets and the sharp alveolar prominences are easily removed with Rongeur forceps preparatory to the final shaping of the ridge. In determining the extent to which the process should be trimmed, the operator is guided by his previous study of the models of the patient's mouth, the observance of malocclusion or relation of the maxillary arch to the mandibular arch, the amount of gum that shows in the act of smiling or talking, but mostly by his previous experience in this branch of oral surgery, and his knowledge of denture construction. Even after a carèful study of the case, very often the operator is face to face with a lack of sufficient, instead of too much, bone and is forced to change his plans during the operation. The frequent occurrence of this predicament can not be too strongly emphasized as the greatest danger in this work.

The alveolar ridge has now taken on a more or less definite shape, but requires the smoothing of the sharp edges, proper beveling for ridge lap and base plate and the general shaping of the arches.

At this time we should refer to the study models, bearing in mind the typal form of the arch we are operating upon and remembering the following facts regarding physical size: (1) large upper or lower affords the greatest possible advantages for stabilizing the dentures; (2) medium size affords less advantage for stabilizing the denture; (3) small size presents much difficulty in stabilizing dentures, consequently much less efficiency in service.

As a general principle, the alveolar ridges should be leveled on their bearing surface as much as possible, simply rounding lingual margin to remove sharp ridges. The buccal margin should be beveled to accommodate ridge lap of the teeth and eliminate abnormal irregularities.

In Class 1, or the square type, we usually find the ridges very strong in their cuspid or bicuspid regions, and it is necessary to bevel the buccal margins higher at these points than any other typal forms.

In Class 2, or the tapering type, we find it necessary to make the long bevel on the labial ridge, near the median line and also to eliminate much soft tissue from the bearing surface of the tuberosities in order to get a level bearing and greater stabilizing value for dentures. It is in this typal form that we usually find a narrower throat and a greater amount of soft tissue around and between the tuberosities, or in other words more displaceable tissue.

In Class 3, the ovoid type, the long bevel is also usually necessary in the labial ridge near the median line.

Both sides of the arch should be made as symmetrical as possible in order that the artificial teeth may be placed on the ridge in their proper positions.

Smoothing of all margins is accomplished by thick heatless stone, which is drawn up and down over the sharp edges and produces a very smooth result. The stones are preferred to files or curettes as they are easier to manipulate and there is less danger of laceration of soft tissues, this also means less discomfort to the patient and a rapidly healing wound.

All previous consideration and steps in the operation, whether they be for aesthetic or mechanical reasons, are of only secondary importance to the removal of the pathological areas connected with the teeth. This part of the operation is of paramount importance and is one upon which not only the success of the operation rests, but the health and perhaps the life of the patient. It surely commends our most careful and most thorough consideration, as to the method of procedure to adopt in the elimination of these areas and all disorganized tissues.

It has always been an acknowledged fact among oral surgeons that a thorough curettement of any infected areas associated with the teeth is necessary to a successful operation, but only in recent years, in fact the last three or four, has this procedure been accepted by the profession in general. The more universal curettement that is being carried out by the dentists today is perhaps the sequel of the established fact that these areas are or may be foci of infection contributing to, if not the sole cause of, the patient's systemic disturbances. Then too with the rapid advance in the art of roentgenology and its universal use, it has been revealed to the more observing men that without a careful curettage frequently the socket of an extracted tooth has healed, again walling in the infection with a more pronounced recurrence of the patient's systemic affection. Having heard so much about oral focal infection and the many ailments attributed to its presence in the human body, the dentist in his zeal to clear the mouth from any suspicion, has resorted to curettement from its mildest form to the removal of the external walls of all septic sockets to the very root ends, commonly called external alveolectomy.

The question naturally arises, Is curettement of the sockets of all non-vital teeth indicated, and, if not, when, where and to what degree? We know that the danger of an infected socket is not determined by the size of the granuloma but by the virulency of the organisms present, the resistance of the patient, and the tension under which these organisms are working; it therefore seems only logical to assume that a certain amount of curettement is warranted in each case, or at least an exploration of the socket. We must also remember that often roentgenograms fail to show any active periapical trouble, in some cases, but upon operating the opposite condition is revealed; and we find considerable destruction of the surrounding osseous tissues.

The degree of curettement warranted in each infected socket will depend upon the amount of the involvement of the tissues, the location of the abscess, the resistance of the patient, and the virulency of the bacteria present. The latter can only be definitely determined by making a culture, but if the destructive process is rapid, we can feel sure that the organisms present are very virulent and a radical operation is indicated. In such event it seems but wise to perform a radical curettage to remove not only the involved tissues but the apparently healthy underlying structures in order to reduce the amount of diseased tissue and approach as nearly as possible the healthy structures.

If the patient is in a weakened condition it would only be fair to him to perform a thorough curettage of all sockets that retained non-vital teeth, resorting to the external alveolectomy in cases where the outer layer of cortical bone is involved close to the gingival, or any other case where necessary in order to establish proper drainage. However, in some of these cases of very low resistance, removal of too much tissue may tax the reparative powers of the patient more than if nature is left to care for the small amount of infection that may have been missed otherwise, and in such case the patient will have to rely on the surgeon's good judgment.

At the time of the preliminary examination of the patient, careful thought and consideration should also be given to the removal of all pathology involving the oral tissues. With a set of good detailed roentgenograms of the affected parts and a careful examination of the mouth, the operator can determine in a large percentage of cases the extent of pathology and the conditions he may expect to find on operating. However, we must remember that the roentgenograms are not infallible and the findings corroborated in each case by a careful exploration of the sockets after the extraction of a tooth should govern the method of procedure. Let the sense of touch, such as relied on in prophylactic technic in the mouth, and a very small curette be the guide. We must not forget to carefully inspect each tooth when extracted, for often the granuloma is removed with it and curettement is then unnecessary. The curettage should be done immediately following the extraction of each non-vital tooth. The roentgenograms should be held to the light by an assistant or placed upon a mount so that the operator can see them during the operation. Curettement of each tooth socket should always be started with a very small curette, one that is capable of reaching the very apex of the socket. In the small abscesses this size of instrument will suffice, but, if not, a larger one can be used. The mistake is very often made of using a curette that is too large, and the seat of infection is not reached.

In cases where roentgenograms show only a small rarefied area at the apex, and upon the removal of the tooth, the end of the root is denuded, we can expect to find, upon exploration, a small granuloma. Upon exploring with a small curette, no larger than the width of the apex of the tooth root, a small cushion-like object is found, which can be easily removed by gentle scraping and teasing down the socket. Whenever this cystic membrane of any size is found at the root end, it is only necessary to remove this soft tissue and not curette the osseous tissues beyond. If necrosis has occurred, curettement of the bony walls is indicated with paralleling of the walls to establish drainage in cases where the destruction of tissue is greater than the diameter of the tooth socket.

If the outer layer of cortical bone is involved, this will be exposed in the retraction of the muco-periosteal flap or by exploration, and will also be a guide in deciding the degree of curettement necessary. In such cases it is advisable to remove the outer wall of the socket providing the gingival third of the plate is involved, but only in such cases does such a radical operation seem warranted. If the gingival third of the alveolar plate is not involved, it is advisable to remove only the portion at the apical third of that tissue which appears involved, allowing the gingival margin, which is in good shape, to remain. In this way the contour of the arch is retained by this bridge of bone and a good foundation is left, over which there will be regeneration of the lost tissue. Nevertheless the fact that some operators advocate the removal of the outer alveolar plate, together with the radical removal of considerable of the circumference of the involved area, with the belief that they are eliminating all infection is very illogical; but it has been proved in a number of research laboratories that the bacteria have penetrated as far as one-quarter of an inch into what appears and is thought to be normal osseous tissue. To date there is not enough convincing evidence to deem it necessary to remove the entire buccal or labial plate of the alveolar ridge to the apex of each and every infected root socket in order that all pathological areas may be eliminated.

In third degree pyorrhetic cases it is essential to remove the unorganized membrane lining the pyorrhea pockets. This can easily be done by loosening it with a curette and when partially detached from the bony wall by holding with hemostatic forceps and dissecting from the bone and gum festoons. This tissue must be removed if we are to avoid a suppurating socket filled with unorganized tissue that will necessitate a second curettement. Due to the hemorrhage that is always met with in removing this membrane, it is difficult to differentiate between it and the swollen inflamed gum festoons, but when it is all removed the bleeding subsides.

The instrumentation necessary to a thorough curettage of infected sockets and associated areas is a set of straight and right and left spoon curettes of various sizes, scapel, hemostats, surgical burs, and curved bone-excising forceps.

After the shaping of the arches with the heatless stone, the sockets are clogged with bone debris which should be removed by a gentle curettement of each socket. The muco-periosteal flap is then allowed to take its natural position in order to test its length. If it meets the lingual ridge of soft tissues, it is too long, for there may be a formation of soft spongy or scar tissue on the ridge which would interfere with the stabilizing of the dentures. The ideal condition to be had is a space of about 3 mm. between the flaps and the lingual ridge of soft tissues. This affords the formation of new tissue over the crest of the bony ridge, making the denture foundation more stable.

Only in an emergency are cases sutured, because when suturing is omitted (1) the length of the operation is lessened and the shock to the patient decreased and also the unnecessary work for the operator reduced. (2) Open drainage is most desirable. Oft-times a small sequestrum of process will be exfoliated and work to the surface and there will be no unnatural force opposing its exit. Then too it is never necessary to remove a suture to allow drainage, should there be a recurrence of infection or suppuration. (3) In the act of smiling, talking, eating, etc., the muco-periosteal flap and the attached muscles are allowed to take their natural position in relation to the arches when there is no suturing. The most that can be said for suturing in these cases is that it is very spectacular.

The above-described technic is used for operating either maxilla or mandible. Not only in cases of full-mouth extraction preparatory to denture construction should the sharp ridges and prominences of bone be trimmed, but it is our duty to the patient and to our dignified profession to consider the extraction of one, two or more teeth a surgical operation and we should perform it as such, using a technic that will give the best results from a restorative standpoint, that will heal in the shortest space of time, and that will give the most comfort to the patient.

In abnormal edentulous mouths where surgical interference is necessary to successful denture construction, it is essential to follow a thorough preliminary examination such as heretofore described, if we are to correctly diagnose the case. Hypertrophied soft tissue is very often found and it must be removed. The cause for its formation should be eliminated if future recurrence is to be avoided. Frequently very low muscular attachments accompany such cases and these also require resection to avoid tipping of the dentures.

Very often a case presents itself in which a denture can not be worn without considerable discomfort to certain places along the ridge. This usually occurs in the mandible and is caused by a very sharp, knife-edged ridge of bone. Very seldom can this be detected by palpation, but is clearly revealed in the roentgenograms. To relieve this, an incision is made exposing the offending spot and the sharp edge touched with a stone. Healing by first intention usually occurs and the patient can continue wearing the denture with no great discomfort.

I wish to enumerate a few of the many dangers which accompany an operation of this kind: (1) the ease with which too much osseous plate may be removed, thereby loosing retention for the denture; (2) the serious shock that may result from a too lengthy operation or aggravation of the infection in a weak patient; (3) the opening of either or both antra if roentgenograms are not carefully studied (this may easily occur in some edentulous cases where the floor of the maxillary sinus is formed by a very thin maxillary ridge and in those cases of large antra where the floor dips down between the roots of bicuspid and molar teeth); (4) the serious results of hemorrhage in hemophilacs, if the patient is not carefully questioned.

Before dismissal, following the operation, the mouth is irrigated with a 1 per cent. Dakin solution and the patient instructed as to home prophylactic measures. The patient returns to the office as often as necessary for postoperative treatments, which usually consist of irrigation, application of remedies to relieve any pain that may result, and dressing of any sacket in which dressing is to be maintained.

The average case heals in about two weeks and at the end of the third impressions can be taken with practically no irritation.

The results usually accomplished in surgical preparation of the mouth are very gratifying to the dentist as well as to the patient. Not only is the patient benefited by the correction of facial contour, but also systemically by the elimination of absorption of the bacterial toxine, in the removal of the pathological areas, and by his ability to efficiently masticate his food with the properly constructed dentures.

